# P56 D-amino acids: the role of D-leucine in biofilm eradication in *Pseudomonas aeruginosa*

**DOI:** 10.1093/jacamr/dlaf230.063

**Published:** 2025-12-04

**Authors:** Kavya Sahoo, Melissa Grant, David Wyatt, Ayesha Rahman

**Affiliations:** Dentistry School of Health Sciences, College of Medicine and Health, University of Birmingham, Birmingham, UK; Dentistry School of Health Sciences, College of Medicine and Health, University of Birmingham, Birmingham, UK; Aston Particle Technologies Ltd, Aston University, Aston Triangle, Birmingham, UK; Dentistry School of Health Sciences, College of Medicine and Health, University of Birmingham, Birmingham, UK

## Abstract

**Background:**

Pathogens colonizing the lower respiratory tract (LRT) form robust persistent biofilms, that contribute to chronic infections. Recent research shows the growing importance of amino acids’ impact on biofilm eradication. Amino acids, by enhancing physicochemical properties such as drug solubility and disrupting extracellular DNA (eDNA) are promising adjuvants that can help overcome biofilm associated AMR.^1^

**Objectives:**

In this study, D-leucine has been evaluated for its anti-biofilm activity against monomicrobial and polymicrobial biofilms of *Pseudomonas aeruginosa*. The synergistic interactions between D-leucine and amikacin against monomicrobial microbes were comprehended.

**Methods:**

The potential of this amino acid was assessed using MIC assay, chequerboard assay, crystal violet staining and confocal fluorescence live/dead assay imaging techniques.

**Results:**

D-leucine demonstrated significant biofilm inhibition by up to 63.3% reduction in density as compared to the untreated control biofilm at a concentration of 40 mM. Confocal fluorescence laser scanning microscopy (CLSM) confirmed these insights by showing an up to 58% reduction in the treated biofilm thickness as compared to the control. Upon investigating the potential synergy of D-leucine and amikacin (defined as FICI index ≤0.5, with growth suppressive at and below OD_600_ of 0.1 for PA01 and 0.6 for PA14), the effective antibiotic concentrations lowered by 2–4 fold when compared to their individual MICs.

**Conclusions:**

These results can be explained by D-leucine potentially substituting the position of L-leucine in leucine aminopeptidase, an enzyme that is essential for biofilm formation, and as a result slowing or inhibiting biofilm development.^3^ Additionally, mutations in the *dtd* gene (deaminoacyl-tRNA deacylase) in *P. aeruginosa* PAO1, which prevents incorporation of D-amino acids into proteins, could allow D-amino acids to be mistakenly incorporated in place of L-amino acids, further contributing to its antibiofilm activity.^4^ Overall, these findings highlight D-leucine as a promising antibiotic adjuvant to new treatment regimens against biofilm associated AMR.
